# Supplementary material for: The Dual Prey-Inactivation Strategy of Spiders—In-Depth Venomic Analysis of Cupiennius salei
Source: Toxins (Basel). 2019 Mar 19;11(3):167. doi: 10.3390/toxins11030167 (PMC6468893; doi:10.3390/toxins11030167)
Supplement: Supplementary file 1 [file toxins-11-00167-s001.zip › Supplementary Fig S1.1-S1.19/Supplementary Fig S1.3.pdf]

Supplementary Fig S1.3

CLUSTAL O(1.2.4) multiple sequence alignment: Venom serine proteases 1 and 2

```
tr_A0A2I5YNR5_CUPSA      --MGLILVTFLLLL-----TGYSSGKLEPVKGCGKALV-PQGRIVNGNVATRGKYPWMVS
tr_A0A2I5YNR1_CUPSA      --MGLILVTFLLLL-----TGYSSGKLEPVKGCGKALV-PQGRIVNGNVATRGKYPWMVS
VSP1_a1_S2_CUPSA         --MGLILVTFLLLL-----TGYSSGKLEPVKGCGKALV-PQGRIVNGNVATRGKYPWMVS
VSP1_b1_CUPSA            --MGLILVTFLLLL-----TGYSSGKLEPVKGCGKALV-PQGRIVNGNVATRGKYPWMVS
VSP1_b2_CUPSA            --MGLILVTFLLLL-----TGYSSGKLEPVKGCGKALV-PQGRIVNGNVATRGKYPWMVS
VSP2_S1_CUPSA            MIAAICVLCCLQLIRGIPLEQISEADTRPLCPCGDDDSSLNTRVVGGHAASRHQFPYASS
                            .: ::  * *:        *... .*:  **.     : *:*.*:.*:* ::*:  *

tr_A0A2I5YNR5_CUPSA      IHE--KIGS-------KMQQACGGAILNENWIVTAAHCIDQPINLNNYEIYAGLFSILKK
tr_A0A2I5YNR1_CUPSA      IHE--KIGS-------KMQQACGGAILNENWIVTAAHCIDQPINLNNYEIYAGLYSLLKK
VSP1_a1_S2_CUPSA         IHE--KIGS-------KMQQACGGAILNENWIVTAAHCIDQPINLNNYEIYAGLFSILKK
VSP1_b1_CUPSA            IQE--KIGD-------KMQHICGGAILNETWIVTAAHCFDQPINLTDYEVYAGLFSILKK
VSP1_b2_CUPSA            IQE--KIGD-------KMQHICGGAILNETWIVTAAHCFDQPINLTDYEVYAGLFSILKK
VSP2_S1_CUPSA            ILFRPHFGDRELREFIRSTPFCGGTLITDRHVITAAHCLKERFPE-DIAVDVGDYSLKEK
                         *    ::*.       :    ***:::.:  ::*****:.: :   :  : .* :*: :*

tr_A0A2I5YNR5_CUPSA      TEPTVQKLQLAKVIIHDKYDEDGLTNDIALIKTATPIDIKGSKGYVNAICLPPGLT-NPT
tr_A0A2I5YNR1_CUPSA      TEPTVQKLQLAKVIIHDKYDEDGLTNDIALIKTATPIDIKGSKGYVNAICLPPGLT-NPT
VSP1_a1_S2_CUPSA         TEPTVQKLQLAKVIIHDKYDEDGLTNDIALIKTATPIDIKGSKGYVNAICLPPGLT-NPT
VSP1_b1_CUPSA            TEPTVQKLQLEKVIIHDKYDKDGLTNDIALIKTATPIDIKGSKGYVNAICLPPGLT-NPT
VSP1_b2_CUPSA            TEPTVQKLQLEKVIIHDKYDKDGLTNDIALIKTATPIDIKGSKGYVNAICLPPGLT-NPT
VSP2_S1_CUPSA            RG--KQILNSRNLTRFPEYIKTSFHTDIAIVELEHPVQFRS--GLMTARLPPPDLDLKPG
                              * *:  ::  . :* : .: .***:::   *::::.  * :..   **.*  :*

tr_A0A2I5YNR5_CUPSA      GEATVIGWGMLRDDGPISAELREVQVPIVPWANCKKIYGDKN-SEFDFVQVVPSMLCAGG
tr_A0A2I5YNR1_CUPSA      GEATVIGWGMLRDNGPISAELREVQVPIVPWANCKKIYGDKN-SEFDFVQVVPSMLCAGG
VSP1_a1_S2_CUPSA         GEATVIGWGMLRDDGPISAELREVQVPIVPWANCKKIYGDKN-SEFDFVQVVPSMLCAGG
VSP1_b1_CUPSA            GEATVIGWGMIRDDGPISAELREVQVPIVPWATCKKIYGDKNNSEFDFVQVVPSMLCAGG
VSP1_b2_CUPSA            GEATVIGWGMIRDDGPISAELREVQVPIVPWATCKKIYGDKNNSEFDFVQVVPSMLRAGG
VSP2_S1_CUPSA            TTVSVYGWGRLSYYGDHPDVLNSVDLPVVDSKQCQPKFV---------SRIEPAMICAGG
                           .:* *** :   *     *..*::*:*    *:  :           :: *:*: ***

tr_A0A2I5YNR5_CUPSA      N-GKDSCQYDSGGPLFQYDKNGVATLIGTVASGGDCGHGPYPGMYMKVSAFKSWMDKVTT
tr_A0A2I5YNR1_CUPSA      N-GKDSCQYDSGGPLFQYDKNGVATLIGTVASGGDCGHGPYPGMYMKVSAFKSWMDKVTT
VSP1_a1_S2_CUPSA         N-GKDSCQYDSGGPLFQYDKNGVATLIGTVASGGDCGHGPYPGMYMKVSAFKSWMDKVTT
VSP1_b1_CUPSA            N-GKDSCQYDSGGPLFQYDKDDVATLIGTIASGGDCGHGPYPGMYMKVSAFKSWMDKVTT
VSP1_b2_CUPSA            N-GKDSCQYDSGGPLFQYDKDDVATLIGTIASGGDCGHGPYPGMYMKVSAFKSWMDKVTT
VSP2_S1_CUPSA            QKGKDACIGDSGSGLV-VRLDNKFVLCGVVSFGRRCALPDVPGVYTRVSSFVDWILKQTQ
                         : ***:*  ***. *.    :.  .* *.:: *  *.    **:* :**:* .*: * *

tr_A0A2I5YNR5_CUPSA      ------------
tr_A0A2I5YNR1_CUPSA      ------------
VSP1_a1_S2_CUPSA         ------------
VSP1_b1_CUPSA            ------------
VSP1_b2_CUPSA            ------------
VSP2_S1_CUPSA            SASCRPCVYGKS
```

Percent Identity Matrix - created by Clustal2.1

1: tr_A0A2I5YNR5_CUPSA	100.00	98.94	94.33	93.97	29.10
2: tr_A0A2I5YNR1_CUPSA	98.94	100.00	93.26	92.91	29.85
3: VSP1_a1_S2_CUPSA	100.00	98.94	94.33	93.97	29.10
4: VSP1_b1_CUPSA	94.33	93.26	100.00	99.65	30.22
5: VSP1_b2_CUPSA	93.97	92.91	99.65	100.00	29.85
6: VSP2_S1_CUPSA	29.10	29.85	30.22	29.85	100.00

Proteomic coverage trypsin VSP1

>tr|A0A2I5YNR5|A0A2I5YNR5_CUPSA PQM protease OS=Cupiennius salei OX=6928 PE=2 SV=1

MGLILVTFLL LLTGYSSGKL EPVKGCGKAL VPQGRIVNGN VATRGKYPWM VSIHEKIGSK MQQACGGAIL NENWIVTAAH
CIDQPINLNN YEIYAGLFSI LKKTEPTVQK LQLAKVIIHD KYDEDGLTND IALIKTATPI DIKGSKGYVN AICLPPGLTN
PTGEATVIGW GMLRDDGPIS AELREVQVPI VPWANCKKIY GDKNSEFDFV QVVPSMLCAG GNGKDSCQYD SGGPLFQYDK
NGVATLIGTV ASGGDCGHGP YPGMYMKVSA FKSWMDKVTT

>tr|A0A2I5YNR1|A0A2I5YNR1_CUPSA PQM protease OS=Cupiennius salei OX=6928 PE=2 SV=1
MGLILVTFLL LLTGYSSGKL EPVKGCGKAL VPQGRIVNGN VATRGKYPWM VSIHEKIGSK MQQACGGAIL NENWIVTAAH
CIDQPINLNN YEIYAGLYSL LKKTEPTVQK LQLAKVIIHD KYDEDGLTND IALIKTATPI DIKGSKGYVN AICLPPGLTN
PTGEATVIGW GMLRDNGPIS AELREVQVPI VPWANCKKIY GDKNSEFDFV QVVPSMLCAG GNGKDSCQYD SGGPLFQYDK
NGVATLIGTV ASGGDCGHGP YPGMYMKVSA FKSWMDKVTT

>VSP1_b1_CUPSA venom serine protease 1
MGLILVTFLL LLTGYSSGKL EPVKGCGKAL VPQGRIVNGN VATRGKYPWM VSIQEKIGDK MQHICGGAIL NETWIVTAAH
CFDQPINLTD YEVYAGLFSI LKKTEPTVQK LQLEKVIIHD KYDKDGLTND IALIKTATPI DIKGSKGYVN AICLPPGLTN
PTGEATVIGW GMIRDDGPIS AELREVQVPI VPWATCKKIY GDKNNSEFDF VQVVPSMLCA GGNGKDSCQY DSGGPLFQYD
KDDVATLIGT IASGGDCGHG PYPGMYMKVS AFKSWMDKVT T

>VSP1_b2_CUPSA venom serine protease 1
MGLILVTFLL LLTGYSSGKL EPVKGCGKAL VPQGRIVNGN VATRGKYPWM VSIQEKIGDK MQHICGGAIL NETWIVTAAH
CFDQPINLTD YEVYAGLFSI LKKTEPTVQK LQLEKVIIHD KYDKDGLTND IALIKTATPI DIKGSKGYVN AICLPPGLTN
PTGEATVIGW GMIRDDGPIS AELREVQVPI VPWATCKKIY GDKNNSEFDF VQVVPSMLRA GGNGKDSCQY DSGGPLFQYD
KDDVATLIGT IASGGDCGHG PYPGMYMKVS AFKSWMDKVT T

Proteomic coverage trypsin VSP2

>VSP2_S1_CUPSA venom serine protease 2
MIAAICVLCC LQLIRGIPLE QISEADTRPL CPCGDDDSSL NTRVVGGHAA SRHQFPYASS ILFRPHFGDR ELREFIRSTP
FCGGTLITDR HVITAAHCLK ERFPEDIAVD VGDYSLKEKR GKQILNSRNL TRFPEYIKTS FHTDIAIVEL EHPVQFRSGL
MTARLPPPDL DLKPGTTVSV YGWGRLSYYG DHPDVLNSVD LPVVDSKQCQ PKFVSRIEPA MICAGGQKGK DACIGDSGSG
LVVRLDNKFV LCGVVSFGRR CALPDVPGVY TRVSSFVDWI LKQTQSASCR PCVYGKS

Proteomic coverage chymotrypsin VSP1

>tr|A0A2I5YNR1|A0A2I5YNR1_CUPSA PQM protease OS=Cupiennius salei OX=6928 PE=2 SV=1
MGLILVTFLL LLTGYSSGKL EPVKGCGKAL VPQGRIVNGN VATRGKYPWM VSIHEKIGSK MQQACGGAIL NENWIVTAAH
CIDQPINLNN YEIYAGLYSL LKKTEPTVQK LQLAKVIIHD KYDEDGLTND IALIKTATPI DIKGSKGYVN AICLPPGLTN
PTGEATVIGW GMLRDNGPIS AELREVQVPI VPWANCKKIY GDKNSEFDFV QVVPSMLCAG GNGKDSCQYD SGGPLFQYDK
NGVATLIGTV ASGGDCGHGP YPGMYMKVSA FKSWMDKVTT

>tr|A0A2I5YNR5|A0A2I5YNR5_CUPSA PQM protease OS=Cupiennius salei OX=6928 PE=2 SV=1
MGLILVTFLL LLTGYSSGKL EPVKGCGKAL VPQGRIVNGN VATRGKYPWM VSIHEKIGSK MQQACGGAIL NENWIVTAAH
CIDQPINLNN YEIYAGLFSI LKKTEPTVQK LQLAKVIIHD KYDEDGLTND IALIKTATPI DIKGSKGYVN AICLPPGLTN
PTGEATVIGW GMLRDDGPIS AELREVQVPI VPWANCKKIY GDKNSEFDFV QVVPSMLCAG GNGKDSCQYD SGGPLFQYDK
NGVATLIGTV ASGGDCGHGP YPGMYMKVSA FKSWMDKVTT

>VSP1_b1_CUPSA venom serine protease 1
MGLILVTFLL LLTGYSSGKL EPVKGCGKAL VPQGRIVNGN VATRGKYPWM VSIQEKIGDK MQHICGGAIL NETWIVTAAH
CFDQPINLTD YEVYAGLFSI LKKTEPTVQK LQLEKVIIHD KYDKDGLTND IALIKTATPI DIKGSKGYVN AICLPPGLTN
PTGEATVIGW GMIRDDGPIS AELREVQVPI VPWATCKKIY GDKNNSEFDF VQVVPSMLCA GGNGKDSCQY DSGGPLFQYD
KDDVATLIGT IASGGDCGHG PYPGMYMKVS AFKSWMDKVT T

>VSP1_b2_CUPSA venom serine protease 1
MGLILVTFLL LLTGYSSGKL EPVKGCGKAL VPQGRIVNGN VATRGKYPWM VSIQEKIGDK MQHICGGAIL NETWIVTAAH
CFDQPINLTD YEVYAGLFSI LKKTEPTVQK LQLEKVIIHD KYDKDGLTND IALIKTATPI DIKGSKGYVN AICLPPGLTN

PTGEATVIGW GMIRDDGPIS AELREVQVPI VPWATCKKIY GDKNNSEFDF VQVVPSMLRA GGNGKDSCQY DSGGPLFQYD KDDVATLIGT IASGGDCGHG PYPGMYMKVS AFKSWMDKVT T

Proteomic coverage chymotrypsin VSP2

>VSP2_S1_CUPSA venom serine protease 2
MIAAICVLCC LQLIRGIPLE QISEADTRPL CPCGDDDSSL NTRVVGGHAA SRHQFPYASS ILFRPHFGDR ELREFIRSTP FCGGTLITDR HVITAAHCLK ERFPEDIAVD VGDYSLKEKR GKQILNSRNL TRFPEYIKTS FHTDIAIVEL EHPVQFRSGL MTARLPPPDL DLKPGTTVSV YGWGRLSYYG DHPDVLNSVD LPVVDSKQCQ PKFVSRIEPA MICAGGQKGK DACIGDSGSG LVVRLDNKFV LCGVVSFGRR CALPDVPGVY TRVSSFVDWI LKQTQSASCR PCVYGKS

Supplementary Fig S1.3 Multiple sequence alignment of isoforms of VSP1_a (tr_A0A2I5YNR5_CUPSA; tr_A0A2I5YNR1_CUPSA; VSP_a1_S2_CUPSA [MH766620]), VSP1_b (VSP1_b1_CUPSA [MH766621]; VSP1_b2_CUPSA [MH766622]), and VSP2 from *C. salei* (VSP2_S1_CUPSA [MH766623], VSP2_S2_CUPSA [MH766624]; VSP2_S3_CUPSA [MH766625]). Signal peptides are marked in black characters, cysteines in white with a black box, positively charged amino acids in pink, negatively charged amino acids in blue, hydrophobic amino acids in red and polar amino acids in green. The catalytic triad is highlighted in gray, the involved amino acid residues of the proposed active site binding structure are highlighted in yellow. The processing site to obtain the proposed activated serine protease is highlighted in red and the Gly that is used for C-terminal amidation of the light chain is highlighted in blue.
